# Association of social factors and health conditions with capacity and performance

**DOI:** 10.11606/s1518-8787.2022056004152

**Published:** 2022-06-20

**Authors:** Marina Carvalho Arruda Barreto, Ricardo Cartes-Velásquez, Valeria Campos, Larissa Fortunato Araújo, Shamyr Sulyvan de Castro

**Affiliations:** I Universidade Federal do Ceará Faculdade de Medicina Programa de Pós-Graduação em Saúde Pública Fortaleza Ceará Brasil Universidade Federal do Ceará. Faculdade de Medicina. Programa de Pós-Graduação em Saúde Pública. Fortaleza, Ceará, Brasil; II Universidad Andrés Bello Facultad de Odontología Concepción Chile Universidad Andrés Bello. Facultad de Odontología. Concepción, Chile; III Universidad Autónoma de Chile Escuela de Salud Pública Santiago de Chile Chile Universidad Autónoma de Chile. Escuela de Salud Pública. Santiago de Chile, Chile

**Keywords:** Disability Evaluation, Health Status, Risk Factors, Socioeconomic Factors

## Abstract

**OBJECTIVE:**

Determine and measure the association of social factors and health conditions with worse capacity and performance levels.

**METHODS:**

Dependent variables consisted of performance and capacity; independent variables comprised age, gender, level of education, personal income, and health conditions. Means (95%CI) of performance and capacity were presented according to the independent variables. Generalized linear models, using a mutual adjustment for all variables considered statistically significant (p < 0.05), measured the associations between each exposure and outcomes. Study population included 12,265 individuals.

**RESULTS:**

Older women with lower education and income levels and with some health condition showed the worst performance and capacity.

**CONCLUSION:**

Results showed that the capacity and performance levels of the Chilean population changed according to social demographic characteristics and health conditions.

## INTRODUCTION

One of modern society’s fundamental goals is to achieve higher levels of health and well-being in the population^1^. As most non-communicable health conditions are non-lethal, in addition to morbidity and mortality, functioning contributes by measuring the impact of health conditions and the outcome of interventions and health promotion^2^.

Given the increased life expectancy, monitoring the population’s functioning profile would help to understand how health conditions impact the population and their consequences. An effective health approach requires a focus on improving health and functioning rates, and not just disease reduction and control^3^. Besides, strategies for collecting population data on functioning or disability lack some standardization, since different approaches are adopted^4^. Considering these issues and exercising its role as a supporting agent in the process of collecting and generating health data, the World Health Organization (WHO) designed and launched the Model Disability Survey (MDS) - a tool for collecting populational data on disability based on the biopsychosocial approach recommended by the International Classification of Functioning, Disability and Health (ICF), which expresses disability as a universal phenomenon^5^. The MDS operationalizes the concept of disability by adopting two perspectives: capacity and performance^3^. The ICF defines disability as “an umbrella term for impairments, activity limitations and participation restrictions. It denotes the negative aspects of the interaction between an individual (with a health condition) and that individual’s contextual factors (environmental and personal factors)” , and capacity as “a construct that indicates, as a qualifier, the highest probable level of functioning that a person may reach in a domain in the activities and participation list at a given moment. Capacity is measured in a uniform or standard environment, and thus reflects the environmentally adjusted ability of the individual”. Still according to the ICF, performance “is a construct that describes, as a qualifier, what individuals do in their current environment, and so brings in the aspect of a person’s involvement in life situations”^6^. Such background highlights the suitability of MDS for collecting standardized population data on disability and in compliance with WHO recommendations. Moreover, a few countries have already used the MDS to survey population data on disability^7^.

Besides, to our knowledge, no published studies have addressed capacity and performance at the population level with robust samples comprising adult and older adult men and women and using the ICF approach. Thus, this study sought to determine and measure the association of age, gender, income, schooling and health conditions with worse capacity and performance levels in the Chilean population.

## METHODS

### Study Design

This is a cross-sectional study with data from a 2015 survey carried out in Chile – the II National Disability Survey (II Estudio Nacional de la Discapacidad – II-ENDISC)^9^.

### Sample

The II-ENDISC is a national household survey conducted with civilians aged 2 to 17 and older, funded by the Ministry of Social and Family Development. Data collection took place in 2015 by means of interviews with 17,780 people selected by probabilistic sampling in urban and rural areas. The present study used data from individuals over 17 years of age (n = 12,265)^11^.

### Outcome Measures

Our primary research outcomes comprised performance and capacity, both expressed by scores ranging from 0 (best) to 100 (worst). These variables were extracted from the MDS functioning block, which includes questions on mobility, hand and arm use, self-care, seeing, hearing, pain, energy and drive, breathing, affect (depression and anxiety), interpersonal relationships, handling stress, communication, cognition, household tasks, community and citizenship participation, caring for others, and work and schooling^5^. The survey is answered by a household informant, gathering data via the following sections: socio-demographic characteristics; work history and benefits; environmental factors; functioning; health conditions and capacity; health care utilization; satisfaction, personality and well-being^5^.

Based on the scientific literature on disability, we selected the following self-reported exposures: age (< 38; 38–57; 58–77; and > 78 years old), gender (men/women), level of education (none; incomplete primary education; complete primary; incomplete secondary education; complete secondary; incomplete tertiary education; and complete tertiary), personal income (categorized into quintiles)^12^, and health conditions (hypertension^13^; arthrosis; chronic respiratory disease^14^; low back pain (LBP)^14^; migraine^14^; anxiety^14^; dementia^15^; chronic kidney disease (CKD)^14^; rheumatic diseases^16^; and sleep disorders^17^).

### Statistical Analyses

Study population characteristics were presented using means or frequencies and their respective 95% confidence interval (95%CI). Means and their respective 95%CI of performance and capacity were presented by social demographic characteristics and health conditions.

We used a generalized linear model (GLM) with logarithmic linkage and gamma distribution to measure differences between categories of outcome explanatory variables due to variable distribution. Our choice of a GLM instead of a linear regression model with log-transformed response was due to the simplicity and greater interpretative understanding of GLM parameters. GLM uses the arithmetic means ratio (AMR) and its respective 95%CI to compare categories of the same response variable and differences are interpreted as percentages (p-value < 0.05).

Weighted sampling was calculated using the survey module (svy command) of the Stata program.

## RESULTS

In our study population (n = 12,265), the mean age was 46.47 years (95%CI: 45.98–46.96). Mean capacity score was 27.13 (95%CI: 26.67–27.56), while performance was 34.14 (95%CI: 33.65–34.64).

Table 1 summarizes the socio-demographic characteristics and health conditions of the sample. The study population comprised mainly people under 58 years of age with an intermediate educational level, a slightly higher number of women, and frequent reports of the following health conditions: hypertension, LBP, arthrosis, and headache.

**Table 1:** Characteristics of the study population (II ENDISC, 2015).

Variables	n (12,265)	%	95%CI
Age group (years)			
< 38	3,955	35.36	34.07–36.67
38–57	4,373	36.41	35.22–37.61
58–77	3,125	22.88	21.86–23.93
> 77	812	4.84	4.84–5.89
Gender			
Male	5,307	48.28	47.18–49.39
Female	6,958	51.71	50.60–52.81
Level of education			
Complete tertiary	2,138	17.81	16.62–19.06
Incomplete tertiary	1,293	12.68	11.77–13.62
Complete secondary	3,412	28.04	26.93–29.19
Incomplete secondary	1,760	14.15	13.29–15.06
Complete primary	1,414	11.05	10.20–11.95
Incomplete primary	1,906	13.73	12.86–14.65
Illiterate	337	2.53	2.19–2.91
Income (quintiles)			
V (Best)	2,199	18.19	16.97 – 19.49
IV	2,409	20.77	19.73 – 21.85
III	2,508	21.25	20.23 – 22.31
II	2,559	20.86	19.73 – 22.03
I (Worse)	2,590	18.91	17.96 – 19.89
Chronic diseases			
Hypertension	3,378	26.34	25.27 – 27.44
Arthrosis	2,181	16.70	15.75 – 17.70
Respiratory diseases	519	3.74	3.30 – 4.22
Low back pain	2,737	22.06	21.02 – 23.13
Migrane	1,981	15.89	15.01 – 16.80
Anxiety	1,633	12.85	11.99 – 13.76
Dementia	111	0.78	0.62 – 0.99
Chronic kidney disease	291	2.30	1.95 – 2.71
Rheumatic disease	224	1.59	1.32 – 1.91
Cerebral palsy	19	0.15	0.08 – 0.26
Sleep disorders	1,448	11.19	10.38 – 12.04

95%CI: 95% confidence interval.

Table 2 shows the means for capacity and performance scores by sociodemographic characteristics and health conditions. Older women with low education and income levels and with any of the health conditions studied had the worse overall capacity and performance.

**Table 2:** Description of capacity and performance scores by sociodemographic characteristics and health conditions (II ENDISC, 2015).

Variables	Capacity (n = 12,265)		Performance (n = 12,265)	
	Mean	95%CI	Mean	95%CI
Age group (years)				
< 38	19.77	19.13–20.41	23.21	27.46–28.95
38–57	27.24	26.52–27.96	34.25	33.52–34.98
58–77	34.13	33.28–34.99	39.81	38.95–40.67
> 77	45.11	43.52–46.70	48.47	47.17–49.76
Gender				
Male	23.75	23.11–24.39	31.03	30.35–31.72
Female	30.29	26.67–30.91	37.05	36.45–37.65
Level of education				
Complete tertiary	21.63	20.70–22.56	28.72	27.64–29.79
Incomplete tertiary	21.13	20.00–22.25	29.10	27.70–30.51
Complete secondary	25.46	24.72–26.21	32.58	31.75–33.40
Incomplete secondary	28.09	27.00–29.17	35.69	34.71–36.66
Complete primary	31.60	30.21–32.98	37.82	36.59–39.05
Incomplete primary	35.17	34.09–36.26	41.83	40.84–42.82
Illiterate	45.80	43.33–48.26	48.49	46.50–50.49
Income (quintiles)				
V (Best)	22.58	21.58–23.57	29.23	28.10–30.36
IV	25.74	24.86–26.63	32.74	31.73–33.75
III	27.04	26.07–28.01	34.28	33.30–35.27
II	30.03	29.06–30.99	36.91	36.04–37.78
I (Worse)	29.94	29.00–30.87	37.20	36.29–38.11
Hypertension				
No	23.67	23.17–24.16	31.24	30.68–31.80
Yes	36.81	36.01–37.62	42.26	41.54–42.99
Arthrosis				
No	24.18	23.75–24.61	31.55	31.05–32.06
Yes	41.84	40.91–42.77	47.06	46.40–47.72
Respiratory disease				
No	26.53	26.07–27.00	33.62	33.11–34.13
Yes	42.45	40.11–44.80	47.60	46.03–49.16
Low back pain				
No	24.38	23.90–24.85	31.41	30.88–31.95
Yes	36.86	36.02–37.70	43.80	43.12–44.48
Migrane				
No	25.33	24.84–25.82	32.40	31.88–32.92
Yes	36.65	35.61–37.69	43.36	42.45–44.27
Anxiety				
No	25.41	24.95–25.88	32.42	31.91–32.92
Yes	38.77	37.58–39.97	45.83	44.93–46.74
Dementia				
No	26.86	26.41–27.32	33.95	33.46–34.45
Yes	60.68	57.47–63.89	58.07	55.96–60.17
Chronic kidney disease				
No	26.73	26.27–27.19	33.80	33.30–34.30
Yes	44.03	41.48–46.58	48.67	46.72–50.62
Rheumatic disease				
No	26.85	26.39–27.32	33.89	33.39–34.39
Yes	44.22	41.98–46.46	49.78	48.26–51.31
Cerebral palsy				
No	27.09	26.63–27.55	34.11	33.62–34.61
Yes	53.19	46.16–60.23	54.68	49.44–59.92
Sleep disorders				
No	25.10	24.63–25.57	32.44	31.94–32.95
Yes	43.26	42.31–44.20	47.64	48.80–48.47

Note: high values for capacity and performance scores denote worst outcomes.

Table 3 presents the associations of sociodemographic characteristics and health conditions with capacity and performance. Adjusted GLM analyses showed that people over 77 years of age have a mean 68% higher capacity score and 37% higher performance score when compared to people under 38 years of age; women have a mean 13% higher capacity score and 9% higher performance score when compared to men; illiterate people had an mean 51% higher capacity score and 31% higher performance score when compared to those with higher education level; people at the worst income level had a mean 11% higher capacity score and 11% higher performance score when compared to those at the highest income level. Having dementia resulted in a mean 45% higher capacity score and 25% higher performance score; cerebral palsy showed a mean 71% higher capacity score and 42% higher performance score; LBP presented a mean 25% higher capacity score and 21% higher performance score. Among the health conditions studied, hypertension had the least impact on mean capacity (13%) and performance (8%) scores.

**Table 3:** Associations of sociodemographic characteristics and health conditions with capacity and performance (II ENDISC, 2015).

Variables	Capacity Unadjusted model	Capacity Adjusted model	Performance Unadjusted model	Performance Adjusted model
	AMR (95%CI)	AMR (95%CI)	AMR (95%CI)	AMR (95%CI)
Age group (years)				
< 38	1		1	1
38–57	1.37 (1.32–1.43)	1.20 (1.15–1.25)	1.21 (1.17–1.25)	1.09 (1.05–1.13)
58–77	1.72 (1.66–1.79)	1.37 (1.30–1.44)	1.41 (1.36–1.45)	1.17 (1.12–1.22)
> 77	2.28 (2.17–2.39)	1.68 (1.58–1.80)	1.71 (1.65–1.78)	1.37 (1.30–1.44)
Gender				
Male	1	1	1	1
Female	1.27 (1.23–1.31)	1.13 (1.09–1.17)	1.19 (1.16–1.22)	1.09 (1,06–1.11)
Level of education				
Complete tertiary	1	1	1	1
Incomplete tertiary	0.97 (0.91–1.04)	1.05 (0.98–1.12)	1.01 (0.95–1.07)	1.04 (0.98–1.10)
Complete secondary	1.17 (1.12–1.23)	1.06 (1.00–1.12)	1.13 (1.08–1.18)	1.04 (0.99–1.09)
Incomplete secondary	1.29 (1.22–1.37)	1.10 (1.03–1.17)	1.24 (1.18–1.29)	1.09 (1.04–1.15)
Complete primary	1.46 (1.37–1.55)	1.08 (1.01–1.15)	1.31 (1.25–1.38)	1.06 (1.01–1.12)
Incomplete primary	1.62 (1.54–1.71)	1.17 (1.10–1.25)	1.45 (1.39–1.52)	1.15 (1.10–1.21)
Illiterate	2.11 (1.97–2.27)	1.51 (1.38–1.65)	1.68 (1.59–1.78)	1.31 (1.22–1.40)
Income (quintiles)				
V (Best)	1	1	1	1
IV	1.14 (1.07–1.20)	1.04 (0.98–1.11)	1.12 (1.06–1.17)	1.05 (1.00–1.10)
III	1.19 (1.13–1.26)	1.06 (1.00–1.13)	1.17 (1.11–1.23)	1.07 (1.02–1.13)
II	1.32 (1.26–1.40)	1.12 (1.05–1.19)	1.26 (1.20–1.32)	1.11 (1.06–1.16)
I (Worse)	1.32 (1.25–1.39)	1.11 (1.04–1.18)	1.27 (1.21–1.33)	1.11 (1.06–1.16)
Chronic diseases				
Hypertension	1.55 (1.51–1.60)	1.13 (1.09–1.17)	1.35 (1.32–1.38)	1.08 (1.05–1.11)
Arthrosis	1.73 (1.68–1.77)	1.19 (1.15–1.24)	1.49 (1.46–1.52)	1.15 (1.12–1.17)
Respiratory diseases	1.59 (1.50–1.69)	1.16 (1.10–1.23)	1.41 (1.36–1.46)	1.13 (1.09–1.17)
Low back pain	1.51 (1.47–1.55)	1.25 (1.22–1.29)	1.39 (1.36–1.42)	1.21 (1.18–1.24)
Migrane	1.44 (1.39–1.49)	1.22 (1.18–1.26)	1.33 (1.30–1.37)	1.16 (1.35–1.19)
Anxiety	1.52 (1.47–1.58)	1.28 (1.23–1.33)	1.41 (1.38–1.44)	1.24 (1.21–1.28)
Dementia	2.25 (2.13–2.38)	1.45 (1.34–1.57)	1.71 (1.64–1.77)	1.25 (1.18–1.33)
Chronic kidney disease	1.64 (1.55–1.74)	1.20 (1.19–1.28)	1.43 (1.38–1.50)	1.13 (1.08–1.19)
Rheumatic disease	1.64 (1.56–1.73)	1.20 (1.12–1.29)	1.46 (1.42–1.51)	1.14 (1.09–1.20)
Cerebral palsy	1.96 (1.71–2.24)	1.71 (1.33– 2.20)	1.60 (1.45–1.76)	1.42 (1.21–1.66)
Sleep disorders	1.72 (1.67–1.77)	1.30 (1.26–1.34)	1.46 (1.43–1.50)	1.17 (1.14–1.20)

95%CI: 95% confidence interval; AMR: arithmetic mean ratio.

## DISCUSSION

This study highlights that being an older women with low education and income levels, and having hypertension, arthrosis, respiratory diseases, LBP, headache, anxiety, dementia, CKD, rheumatic diseases, cerebral palsy and sleep disorders were associated with worst capacity and performance.

According to the ICF, personal factors influence functioning and are therefore paramount for understanding health and functioning profiles^6^. Similarly, gender, level of education and personal income are considered social determinants of health^3^ and have reported influence on the occurrence of disabilities^18,19^. Regarding the Chilean population under study, we found that women had the worst capacity and performance, which generally worsened as education and income decreased. These findings further emphasize the relationship between poverty and disability (expressed here by capacity and performance), emphasizing that disabilities can stem from poverty and vice versa, placing the person with disability in a vicious cycle where poverty leads to more disability, which leads to more poverty^20^. Hence, not only health policies but also social policies can positively impact the population’s functioning. Since research on disability has consistently shown worse disability rates among women worldwide^18^, policies aimed at providing social protection and health services to women could also be effective in improving disability rates in the population. This study also confirmed the association between the aging process and worsening functioning^19^, expressed here by the directly proportional increase in the mean capacity and performance scores with age.

Interviewees with health conditions showed deteriorated capacity and performance. Research shows that hypertension is associated with disabilities due, in part, to behavioural risk factors such as obesity, smoking, and physical inactivity^21^. Cognitive mechanisms may also be involved in the occurrence of disabilities in hypertensive individuals, since cognitive impairment could lead to less than optimal or poor lifelong decisions, allowing for the appearance or worsening of disabilities^22^.

Arthritis/arthrosis, in turn, implies limitations resulting from pain^23^. Disability is also associate with respiratory diseases, possibly due to muscle strength, lower extremity function, exercise performance, and mobility-related dyspnea^24^. LBP is associated with limitation of activities such as personal hygiene, household chores, and carrying shopping bags due to movement limitations^25^. Migraine is also associated with disabilities, probably caused by pain intensity, attack duration, and number of migraine symptoms^26^. The association between anxiety and disability may be explained by the anxiety response caused by exposure to diverse social situations, panic attacks, and agoraphobia^27^.

Moreover, individuals with dementia are more likely to develop disabilities than others, as this health condition is highly stigmatizing and affects cognitive and independent living skills^28^. Besides physical conditions, socioeconomic, environmental, and individual factors, comorbidities and complications are also associated with higher disability rates in individuals with CKD^29^. As for rheumatic diseases, being an older women who underwent joint surgery and experience pain is associated with disabilities^30^. Cerebral palsy is a very disabling health condition and probably results from motor impairments caused by brain lesions and environmental factors^24^. Finally, sleep disorders are also a predictor of disabilities and this association seems to be indirectly linked to muscle fatigue, which leads to low levels of physical activity, deregulation of systems such as inflammatory, and increased risk of obesity. Importantly, fatigue, overactive inflammatory processes, and obesity are risk factors for disabilities^31^.

As mentioned above, age, gender, level of education, personal income and health conditions are related to increasing occurrence of disability, but the impact of each variable on capacity and performance remains to be discussed. Those aged 38 to 57, 58 to 77, and 78 to 97 years had, respectively, a 20%, 37%, and 68% higher mean capacity score than those under 38 years of age—remembering that the higher the mean, the worse. The performance scores followed a similar pattern, with means of 9%, 17%, and 37%, respectively. Looking at this variable, we see an increasing gradient between the categories, showing a more consistent relationship, with steadily increasing differences in mean capacity and performance scores when related to the reference category.

In the studied population, women averaged 13% higher than men in capacity scores and 9% higher in performance. Regarding level of education, the most significant association was found among those with incomplete primary education and illiterates, with 17% and 51% higher mean capacity scores and 15% and 31% higher mean performance scores, respectively.

As for income, the means showed little difference between categories. Adopting the highest quintile (V) as reference, we found a higher mean capacity score of 4% for quintile V, 6% for quintile III, 12% for quintile II, and 11% for quintile I. For the performance scores, the values were 5%, 7%, 11%, and 11%, respectively.

Hypertension determined a mean 13% higher capacity score; arthritis/arthrosis, 19%; respiratory diseases, 16%; LBP, 25%; migraine, 22%; anxiety, 28%; dementia, 45%; CKD, 20%; rheumatic diseases, 20%; cerebral palsy , 71%; and sleep disorders, 30%. As for performance scores, the pattern was quite similar with a mean 8% higher for hypertension; 15% for arthritis/arthrosis; 13% for respiratory diseases; 21% for LBP; 16% for migraine; 24% for anxiety; 25% for dementia; 13% for CKD; 14% for rheumatic diseases; 42% for cerebral palsy ; and 17% for sleep disorders.

In practice, determining these associations allows us to estimate the mean capacity and performance scores according to each person’s profile. For example, a 56-year-old illiterate woman with lower income suffering from hypertension and LBP will have a 133% higher mean capacity score and 89% higher mean performance score than a 35-year-old man with higher income and no comorbidities. Moreover, such data could be used as a strategy to check the profiles with the highest risk of worsening capacity and performance, pointing out the elements or variables that matter most in worsening functioning.

Although restricted to a representative sample from one country (Chile), this study stands out for bringing to light the discussion about the impact social determinants of health have on capacity and performance. This debate is worldwide and, as the results found here show, should also be applied to the field of disability studies. Would social variables have the same impact on the health of individuals with and without disabilities? Could interventions that seek to address social inequalities be the same for individuals with and without disabilities? These are some of the questions that arise when we verify associations of social variables with worse capacity and performance scores and that should be addressed in future studies.

Moreover, the health conditions studied present an interesting perspective of their association with lower levels of capacity and performance. After adjustments in the statistical analysis process, dementia and cerebral palsy had the highest impact while hypertension proved to be the health condition with the lowest impact among those under study. Our findings stand out for outlining the profile of the population groups that would most need health care aimed at improving capacity and performance. This could have a major individual and collective impact by producing health services and policies targeting these population groups, using interventions designed specifically for such health conditions. The influence of other health conditions on capacity and performance could also be the target of interventions, but without the same urgency and scope as dementia and cerebral palsy.

Although self-reported health conditions are the main limitation of this study, the scientific literature has already discussed the value and importance of collecting self-reported population data^32^. Conversely, its strengths are fivefold. The first and most innovative concerns the quantitative approach to the impact that the studied variables have on capacity and performance. Second, functioning was studied according to definitions established by the WHO. Third, capacity and performance were self-reported, meaning that the collected measurements are person-centered. Fourth, the lack of literature on capacity and performance at the populational level highlights a gap in scientific information that this article hopes to bridge. Lastly, the database derives from a population survey with a complex and reliable sample specifically designed to verify the proposed objectives.

The present study also addresses Article-31 of the Convention on the Rights of Persons with Disabilities (CRPD) regarding statistics and data collection, providing population data collection that enables the formulation and implementation of policies aimed at ensuring the CRPD^33^. Moreover, our research also addresses some of the Sustainable Development Goals (SDG) of the United Nations 2030 Agenda for Sustainable Development^34^ that may be directly relevant to people with disabilities. Goal 4 – ensure inclusive and equitable quality education and promote lifelong learning opportunities for all: since our study confirms that people with lower educational level have compromised capacity and performance, the need for policies targeting education inequality is more evident. Goal 5—achieve gender equality and empower all women and girls—is directly related to our results, considering that women had the worse results. Issues involving income are relevant since lower income implies worse results and Goal 8 posits to “promote sustained, inclusive and sustainable economic growth, full and productive employment and decent work for all.” Our results clearly present inequalities according to the studied variables, which dialogues with Goal 10: “Reduce inequality within and among countries.” These arguments highlight the importance of this study from both a local (the country) and global perspective, as it confirms and reinforces what the United Nations presents as goals for the associated countries via the SDGs. Collection and production of population data on disability using MDS is in line with both the CRPD and the SDGs. Its use has already been reported in the literature and should be reinforced and encouraged^8^.

## CONCLUSION

The capacity and performance of the Chilean population varied with age, gender, level of education, income, and self-reported health conditions or chronic diseases. Older women with low income and educational levels were associated with worse capacity and performance scores. Dementia and cerebral palsy were the health conditions that most affected the outcomes studied.

Overall, our results reinforce that areas such as education, income, gender equality and social inequalities, already established by the United Nations as priorities for global sustainable development in the associated countries, should also be the target of specific policies aimed at improving the living and health conditions of persons with disabilities.
